# Intermittent hypoxia modulates redox homeostasis, lipid metabolism associated inflammatory processes and redox post-translational modifications: Benefits at high altitude

**DOI:** 10.1038/s41598-020-64848-x

**Published:** 2020-05-13

**Authors:** Anamika Gangwar, Subhojit Paul, Yasmin Ahmad, Kalpana Bhargava

**Affiliations:** Peptide and Proteomics Division, Defence Institute of Physiology and Allied Sciences, Defence Research and Development Organization, Lucknow Road, Timarpur, Delhi, 110054 India

**Keywords:** Proteomic analysis, Prognostic markers

## Abstract

Intermittent hypoxia, initially associated with adverse effects of sleep apnea, has now metamorphosed into a module for improved sports performance. The regimen followed for improved sports performance is milder intermittent hypoxic training (IHT) as compared to chronic and severe intermittent hypoxia observed in sleep apnea. Although several studies have indicated the mechanism and enough data on physiological parameters altered by IH is available, proteome perturbations remain largely unknown. Altitude induced hypobaric hypoxia is known to require acclimatization as it causes systemic redox stress and inflammation in humans. In the present study, a short IHT regimen consisting of previously reported physiologically beneficial FIO2 levels of 13.5% and 12% was administered to human subjects. These subjects were then airlifted to altitude of 3500 m and their plasma proteome along with associated redox parameters were analyzed on days 4 and 7 of high altitude stay. We observed that redox stress and associated post-translational modifications, perturbed lipid metabolism and inflammatory signaling were induced by IHT exposure at Baseline. However, this caused activation of antioxidants, energy homeostasis mechanisms and anti-inflammatory responses during subsequent high-altitude exposure. Thus, we propose IHT as a beneficial non-pharmacological intervention that benefits individuals venturing to high altitude areas.

## Introduction

Intermittent hypoxia (IH) is defined as periodic alternating exposures to hypoxia and normoxia. IH has been associated with obstructive sleep apnea^1,2^ as well as sports training^3–5^. It is of utmost importance to delineate the effects of IH observed in obstructive sleep apnea from the beneficial aspects observed in sports training^6–8^. IH training (IHT), which is not as severe as the chronic intermittent hypoxia associated with obstructive sleep apnea, has been reported to exert beneficial hematological, vascular, metabolic, and neurological effects in humans suffering from cardiovascular and pulmonary diseases as well as in overweight and obese individuals^9,10^. Training under hypoxic conditions, particularly IHT during a training session, improves hematological and muscle adaptations and this has been used to improve the exercise performance of athletes by sports medicine community^11,12^. As an extant, IHT is simply a modified version of the live high train low philosophy, where an individual spends most of their time residing at high altitude prior to descending during training session. High altitude training, due to the physiological benefits of hypoxia adaptation has been proposed as a beneficial model for improving the endurance performance of humans and has been used by athletes for more than a century^13–16^. Originally Levine *et al*. demonstrated that exposure for >20 h/day to 2,500 m altitude for 4 weeks resulted in an increase in erythrocyte volume, an increase in VO_2_ max, and improved performance in an event (5,000 m time trial) that was dependent on high rates of oxygen transport^16^. However, contrary to this a control group exposed to identical training at sea level, did not show any improvement in the performance which strengthened the concept of LHTL^7,17,18^ and initiated a quest for understanding the mechanism of altitude training^19^. In the last few years, there has been a remarkable increase in the number of altitude training regimen, both for improving athletic performance and acclimatization for rapid altitude ascent. Nitrogen houses, hypoxia tents, and special breathing apparatuses to provide inspired hypoxia at rest and during exercise and intermittent hypoxia exposures were some of them^7^. Intermittent hypoxia (IH), however has gained recent attention, primarily due to its simplicity and pronounced effects.

The benefits of IH *per se* are also unclear. Although some of the initial studies advocated benefits of IH on health and acclimatization, counterpoints have also been raised^19^. On the positive side, Hinckson *et al*. have shown that altitude exposure simulated with hypoxic tents is likely to enhance performance substantially in middle-distance endurance running events^20^. Dale *et al*., have suggested two unexpected and only recently recognized benefits of IH i.e. improvement in respiratory and non-respiratory somatic motor function, and increased growth/trophic factor expression in the central nervous system (CNS)^21^. While, on the negative side, evidence from animal models of obstructive sleep apnea (OSA) suggested IH is independently associated with metabolic dysfunction, including dyslipidemia and insulin resistance^22^ and intermittent hypoxemia associated with sleep apnea was shown to increase the risk for hypertension and vascular disease^23^.

In light of the existing scientific information, IH remains a paradox yet to be mechanistically explained. More molecular evidences are needed to decide how IH elicits pathology or physiological enhancement. The degree of protection achieved by IH depends on several factors including time, severity and duration of the hypoxic challenge^24^. Most of physiological studies performed on the effects of IH on human performance analyzed key physiological parameters. However, molecular investigations such as plasma proteome profiling were not thought of in the past. Only scant information of the molecular effects of IHT is available. Wang *et al*. have shown that Chronic intermittent hypoxia disturbs insulin secretion and causes pancreatic injury via the MAPK signaling pathway^25^. On the other hand, opposing views exist on sleep apnea associated IH which is known to increase levels of total cholesterol, LDL and triglycerides^22^. Since any hypoxia is primarily a redox stress as it disturbs oxygen homeostasis, redox post-translational modifications provide an important clue regarding the overall effects of IHT. Redox post-translational modifications are primarily caused by excess ROS/RNS chemically modifying amino acid residues in proteins. Some like carbonylation mostly cause loss of function^26,27^ while others like nitrosylation can be beneficial^28,29^. In an vitro study, we had observed direct cross-talk between nitrosylation and carbonylation^30^.

Recent advancements in proteomics and systems biology enable us to obtain a global overview of the metabolic perturbations, which can then be dissected down to the level of causal molecular events. This study was performed with iTRAQ based quantitative proteomics and exhaustive network analysis of the significant proteins to achieve a detailed insight into the global proteomic changes and their integrated meaning. We tried to answer the existing paradoxical view of intermittent hypoxia in relation to high altitude. Moreover, our study was also designed to understand the effect of intermittent hypoxia at sea level and its after-effects on travelling to high altitudes. We hypothesized that the effects of intermittent hypoxia are prolonged and may extend over a week’s span after the high-altitude ascent, therefore we considered the comparison of post IHT intervention on different time points. Previous study by Angela *et al*. already indicates the importance of dose on effects of hypoxia^31^. To check the effects of dose of hypoxia, we used temporal scale of 4 days and 7 days at altitude after the IHT protocol to evaluate the temporal association of post IHT with high-altitude exposure.

In the present study, we investigated global human plasma proteome changes after a repeated mild normobaric IH exposure (12% FIO2, 4 h for 4 consecutive days). We also investigated proteome level changes of same volunteers after subsequent hypobaric hypoxia challenge (12,500 ft) after 4 and 7 days. A basic assessment of the protein carbonyl and nitrosyl content was also performed to observe if IHT had any effect on redox post-translational modifications profile. Overall, proteins involved in lipid metabolism based energy homeostasis, inflammatory signaling and redox homeostasis were observed to be activated by mild IHT which provided a beneficial effect during high altitude exposure. When compared to a previous study having same time-points of high altitude exposure at similar altitude^32^, the benefits of IHT for faster altitude acclimatization are very clear. IHT also induced protein nitrosylation during subsequent high altitude exposure while reducing nitrosyl levels during training at sea-level. Thus, mild IHT regimen, as a non-pharmacologic intervention prior to high altitude exposures can be a promising avenue.

## Materials and Methods

### Materials

All reagents and chemicals were purchased from Sigma Aldrich (St. Louis, MO, USA) unless specified.

#### Experiment design and sample collection

**Figure 1:**
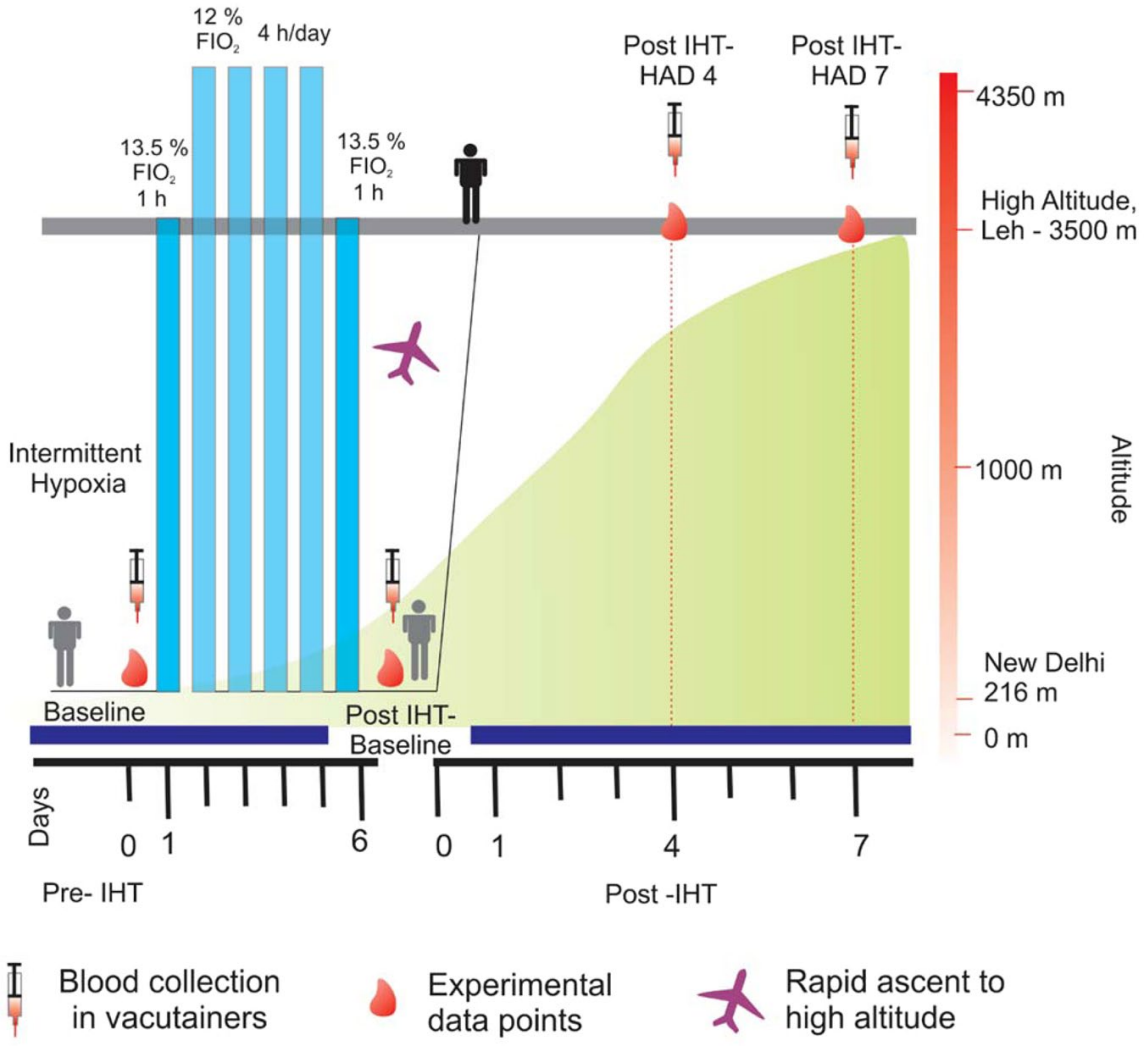
Study design. Blood plasma was harvested from all subjects at Baseline immediately before administering 13.5% FIO_2_ for 1 h (Day 0). Thereafter, subjects underwent exposure to 12% FIO_2_for 4 h a day for 4 days at sea level (Days 1-4). On Day 5, 13.5% FIO_2_ for 1 h was administered and blood sample collected immediately after. All subjects were airlifted to 3500 m. Blood plasma was collected on 4^th^ day and 7^th^ day of high altitude stay, i.e. Post IHT-HAD4 and Post IHT-HAD7.

A total number of 40 male healthy volunteers (age: 22–25 years, height: 170 ± 4 cm, weight: 62 ± 3 kg) were recruited for the present study. Individuals with any kind of medication, smoking and alcohol intake habits as well as high altitude exposure in last one years were excluded. All the study protocols were approved by institutional ethical committee (**I**nstitutional **E**thics **C**ommittee, **D**efence **I**nstitute of **P**hysiology &**A**llied **S**ciences: IEC/DIPAS/09/DIP-251) which is in accordance to Helsinki declaration for human studies and informed written consent was obtained from all the volunteers. The basal parameters of the volunteers were recorded at Delhi (Baseline group) and subjects were exposed to normobaric hypoxia (12% FIO_2_ for 4 h per day; equivalent altitude 4350 m) with pre-hypoxia and post-hypoxia challenges (13.5% FIO_2_ for 1 h; equivalent altitude 3500 m) as described previously^33^. The volunteers were air lifted to Leh (3,520 m) the following day of post-hypoxia challenge and continuously monitored at days 4 (Post IHT-HAD4) and 7 (Post IHT-HAD7) respectively. Fasting venous blood samples were collected in EDTA vacutainers at Delhi (Baseline and Post IHT-Baseline groups) and Leh (Post IHT-HAD4 and Post IHT-HAD7 groups) during morning hour. Study design has been shown in Fig. 1. Plasma was separated by centrifugation at 1000 g for 15 min at 4 °C and stored at -80°C with mammalian protease inhibitor cocktail.

### Quantitative proteomics using iTRAQ

Approximately  100 µL plasma from each individual per experimental group was pooled   to yield four different pools (Baseline, Post IHT-Baseline, Post IHT-HAD4 and Post IHT-HAD7) of 1 ml plasma each. High abundance plasma proteins were depleted using ProteoMiner protein enrichment kit (Cat. no. 163-3006, Bio-Rad, USA) as per the manufacturer’s instructions and protein concentrations were determined by Bradford assay. For iTRAQ labelling, 100 µg of depleted plasma from each group were reduced. The reduced samples were then blocked on cysteines and trypsin-digested overnight (Part # V511QA, Promega, USA) at 37°C as per the manufacturer’s standard procedure for plasma samples. Each pooled plasma sample was labeled with 113 (Baseline), 114 (Post IHT-Baseline), 116 (Post IHT-HAD4) and 118 (Post IHT-HAD7) mass tagged iTRAQ labels (Applied Biosystems, USA). Subsequently, all of the four labeled samples were pooled and fractionated using strong cation exchange (SCX) column. Fifteen top fractions were collected, vacuum dried and reconstituted in 0.1% trifluoroacetic acid. Finally, these samples were desalted. These desalted samples were then subjected to LC-MS/MS analysis using LTQ-Orbitrap Velos mass spectrometer (Thermo Scientific, Germany) coupled with a 1200 nano liquid chromatography system (Agilent Technologies, USA). Data acquisition was accomplished using Xcalibur 2.1. Data dependent acquisition was performed for the spectra. Scans were acquired in Orbitrap mass analyser (mass resolution of 60,000 at m/z 400). The MS/MS data acquisition was done for the top 20 precursor fragments at 15,000 resolution using HID (high energy collision induced dissociation). Unassigned/singly charged precursor ions were rejected. Proteome Discoverer 1.3 (Thermo Scientific, Germany) software was used for MS data analysis. Peptide mass tolerance of ± 10 ppm at MS and 0.1 Da at MS/MS was set during SEQUEST mediated database searches with taxonomy set as *Homo sapiens*. An estimated false positive rate (FDR) was calculated using decoy database search. FDR came as 1% with search parameters having trypsin as protease with one missed cleavage, carbamidomethyl cysteine as a fixed modification, oxidation of methionine as dynamic modification and iTRAQ modifications at N-terminus of the peptide and lysine were set as static modifications.

### Bio-informatics analysis

Proteins were categorized based on their molecular function and subcellular localization. Venn diagram for determination of the overlapping significant proteins identified in the expression data was prepared using online venn diagram tool, venny 2.1 as per the developer’s instructions^34^. The list of significantly regulated proteins was exported in a tab delimited text file and then image was exported in the PNG format at the highest resolution.

### Biological network analysis using IPA

Data was analyzed using Ingenuity pathway analysis (IPA) system^35^ (QIAGEN Inc., https://www.qiagenbioinformatics.com/products/ingenuity-pathway-analysis). IPA was utilized to determine the key canonical pathways, networks and disease functions in the iTRAQ dataset.

All protein IDs were converted to their official symbols using Database for Annotation and Integrated discovery (DAVID, https://david.ncifcrf.gov) and respective fold change ratios were used for determination of canonical pathways and biological process networks. Fold change > 1.5 and p-value < 0.05 was set during all analysis. GO data was overlapped to the findings in IPA. Fisher’s exact test was used to calculate p-value for each of the canonical pathways in the analysis.

### Validation of proteomics data with cytokine array

Cytokine array was performed by using Human XL Cytokine Array Kit (Cat. no.ARY022, R & D systems, USA) as per the manufacturer’s instructions. In brief, array membrane was first blocked in array buffer 6 for one hour on a rocking platform shaker in a 4 well multi-dish. After blocking, the membrane was incubated with desired sample quantity overnight at 2-8 °C on a rocking platform shaker. Then the membrane was washed for 10 min with 1X wash buffer thrice. 1.5 mL per well of diluted detection antibody cocktail was added and incubated for 1 hour on shaker. Then the membrane was again washed with 1X wash buffer thrice. After washing, membrane was incubated with 2 mL of 1X Streptavidin-HRP for 30 mins. Finally, 1 mL of Chemi Reagent mix was spread evenly on each membrane and autoradiograph was developed. Pixel densities on X-ray films were scanned and analysed using a image analysis software (Image J).

### Immunoblotting

Immunoblotting was performed for proteins AMPK (Cat.no.sc-130394,Santa Cruz Biotechnology, UK), PGC (Cat.no.sc-13067,Santa Cruz Biotechnology, UK), ApoA1 (Cat.no.sc-30089, Santa Cruz Biotechnology, UK), Apo B (CSB-PA001918GA01HU, CusaBio, USA), PON1 (Cat no.17A12,Abcam, UK), C1s (Cat.no.HPA018852, Sigma, USA), C-3 (Cat.no.WH0000718M1, Sigma, USA), CRP-1 (Cat.no.40787, SAB, USA), Hif1 alpha (Cat.no.NB100105, Novus Biologicals, USA), Sod 1(Cat.no.sc-8637, santacruz, UK), beta-Actin (Cat. no. A1978, Sigma, USA) using primary antibodies. In brief, 30 µg protein was separated on 10% SDS-PAGE and transferred to PVDF membrane (Cat.no.PI88520, Thermoscientific, USA). The membranes were blocked overnight with 5% Casein blocking buffer (Cat.no.B6429-500mL, Sigma, USA) at 4 °C. After washing thrice with 1X PBST (10 min each), the membranes were incubated with desired dilutions of primary antibodies for 2 hours at room temperature on a rocking platform shaker. Then the membranes were washed thrice and subsequently incubated with respective secondary antibody (1:10,000 dilutions) for 2 h at room temperature on a rocking platform shaker. Finally, the blots were developed using chemiluminescent peroxidase substrate (Cat. no. CPS1300-1KT, Sigma, USA). Images were acquired using gel documentation system (Biospectrum Imaging system, Ultra-voilet products ltd, Cambridge, UK) and analysed using image analysis software (Image J).

### Estimation of protein nitrosylation

The Pierce S-Nitrosylation Western Blot kit (Cat no. 90105, Thermo scientific, USA) was used as per manufacturer’s instructions to estimate protein nitrosyl content in samples. Briefly, 1 M MMTS (2 µl) was added to sample (100 μl) with concentration of 1 μg/μl protein. The mixture was incubated for 30 min at room temperature. Six volumes of pre-chilled acetone were then used for protein precipitation. The precipitate was again resuspended using HENS buffer (100 μl). For each 50 μl sample aliquot, 1 μl labeling reagent was utilized. Further, 2 μl Sodium ascorbate (1 M) was added to the sample followed by incubation of 2 hr at room temperature. Then 10 μl reducing Laemmli sample buffer (5×) was added and heated at 100 °C for 5 min. The samples were then immunoblotted using Anti-TMT antibody (1:1000 in 5% NFDM in 1X TBST) and secondary Anti-mouse IgG-HRP conjugated antibody (1:20,000 in 5% NFDM in 1X TBST). Immunoblots were analyzed using a previously described method^36^.

### Protein oxidation using oxyblot

Oxyblot^TM^ protein oxidation detection kit (Cat no. 7150, Millipore, USA), using manufacturer’s protocol, was utilized to assess protein carbonyl content in plasma. Plasma was aliquoted after protein estimation to ensure equal protein concentration across all groups. 12% SDS (final concentration 6%) was added to 5 μl protein sample followed by immediate addition of 10 μl DNPH and incubation at room temperature for 15 min. Then, 2 μl 5% β-ME was added. Finally, using neutralization solution reaction was terminated. Samples were then immunoblotted (as described under Immunoblotting section) onto nitrocellulose membrane and incubated in anti-DNPH antibody (1:150; 2hrs) and finally with secondary antibody (1:300; 1.5 hr). The blots were developed and images acquired at 300 dpi resolution using E-Gel Imager system (Cat. No. 4466611; Thermo Fisher, USA).

### MCP-1 estimation

ELISA assays were performed for MCP-1 (Cat # E-EL-H0020, Elabscience, USA). Briefly, 100 μ USA). Briefly, 100 μl of plasma sample was added to a pre-coated well and incubated for 90 mins at 37 °C. Then the contents of the well were removed and 100 μl Biotinylated Detection antibody was added. The antibody was also incubated for 1 h at 37 °C. The well was then washed thrice and 100 μl HRP conjugate was added. After incubating for 30 mins at same temperature, the well was again washed thrice. Finally, 90 μl Substrate reagent was added followed by brief incubation of 15 mins and addition of 50 μl Stop solution. Absorbance was read immediately at 450 nm.

### Biochemical Assays

#### ROS estimation

ROS levels was measured by using Carboxy Methyl Dichlorofluorescein diacetate (CM-DCFHDA) (Cat.no.C400, Life Technologies, USA), in plasma of all four groups. We added 150 µl of diluted plasma samples (1:30 in milliQ) with 10 µl of 10 μM CM-DCFHDA and incubated at 37˚C   for 40 minutes in dark. Further, fluorescence was measured using flourometer (LS45 Luminescence Spectrometer, PerkinElmer, Waltham, MA, USA) at 488 nm excitation and 525 nm emission wavelengths, respectively. The data was presented as AU per µl of sample.

### MDA Assay

The Lipid peroxidation status in plasma samples was checked by using method suggested by Ohkawa *et al*.^37^. Briefly, 500 μl  of trichloroacetic acid (TCA; 10% w/v in distilled water) and 500 μl thiobarbituric acid (0.67% w/v in 0.05 M NaOH) were added to 250 μl of the plasma samples, in series, and incubated in a water bath at 95°C for 15 minutes. The mixture was then allowed to cool to room temperature and centrifuged at 400 *g* for 5 minutes. 200  μl of the supernatant from each group was transferred in a 96-well plate in triplicate and optical density was measured at 531 nm using spectrophotometer (VersaMax ELISA Microplate Reader, Molecular Devices, Sunnyvale, CA, USA). The data represented as μmol MDA/mg of protein.

### Total NOx (nitrate + nitrite) levels

Total nitrate and nitrite levels (NOx) in plasma were measured using Nitrate/Nitrite fluorometric assay kit (Cat.no.780051, Cayman Chemical, Ann Arbor, MI, USA) according to manufacturer’s instructions. Briefly, Plasma was filtered through 10 kDa molecular weight cut off microfuge filters. 10 µl of sample or 50 µl of standards were then added in wells of 96 well plate and the volume was adjusted to 100 µl with assay buffer. Further 10µl of DAN reagent was added to each well and incubated for 10 mins. Finally, 10 µl of NaOH was added and fluorescence was measured using fluorometer (LS45 Luminescence Spectrometer, PerkinElmer, Waltham, MA, USA) at excitation wavelength of 360 nm and emission wavelength of 430 nm. The data was presented as as µM of NOx per mg of protein.

### Protein carbonylation

Protein carbonylation colorimetric assay kit (Cat no. K830-100, BioVision, USA) was used to estimate protein carbonyl content following manufacturer’s instructions. To summarize, 100 μl plasma was mixed into 100  µl DNPH. This mixture was then vortexed and incubated for 10 min at room temperature followed by addition of 30 µl TCA. This mixture was then vortexed, incubated on ice (5 min) and centrifuged (13,500 rpm; 2 min). The supernatant was discarded and the pellet retained. The pellet was washed in 500 µl cold acetone, followed by addition of 200 µl Guanidine solution and brief sonication to dissolve it. OD_375_ of 100 µl sample from this solution was measured in a spectrophotometer (Power waveX, Botex Instruments, USA).

### Estimation of Reduced Glutathione

EnzyChrom™ GSH/GSSG Assay kit (Cat. No. EGTT-100, BioAssay Systems, USA) was used to measure total glutathione content in human plasma samples. Briefly, 30 μl plasma samples from each group were deproteinated using 70 μl of 5% wt meta-phosphoric acid. About 15 μl  of clear supernatant is taken and mixed with 485 μl of 1XAssay buffer. Then, 200 μl of the prepared sample per well (ELISA 96-well plate) and 100 μl Working Reagent per well (1X Assay buffer, GR enzyme, NADPH & DTNB) were sequentially added and mixed by tapping gently. Absorbance was taken immediately at zero minute and after 10 min at 412 nm.

### HDL and LDL estimation

HDL and LDL estimation were done using HDL and LDL/VLDL cholesterol assay Kit (Cat# STA-391, Cell Biolabs, Inc., USA) as per manufacturer’s instructions. Briefly, HDL and LDL/VLDL fractions were prepared from plasma samples and added to 96 microtiter plate. 50 µL of the prepared cholesterol reaction reagent was added to each well and the plate was incubated for 45  minutes at 37 °C in dark. The fluorescence was measured with a fluorescence microplate reader (LS45 Luminescence Spectrometer, PerkinElmer, Waltham, MA, USA) equipped for excitation in the 530-570 nm range and for emission in the 590-600 nm range. Finally, the concentration of cholesterol was calculated within samples by comparing the sample RFU to the cholesterol standard curve.

### Statistical analysis

For biochemical assays and ELISA, results were presented as mean value ± standard error of mean (SEM). Graph pad prism (v 5.0) (using 1-way analysis of variance (ANOVA) followed by Bonferroni’s Multiple Comparison test) was used to analyze datasets generated. Significance was set at p < 0.05.

## Results

### Global proteomics analysis and protein networks identified suggest a lipid metabolism derived signaling cascade

**Figure 2:**
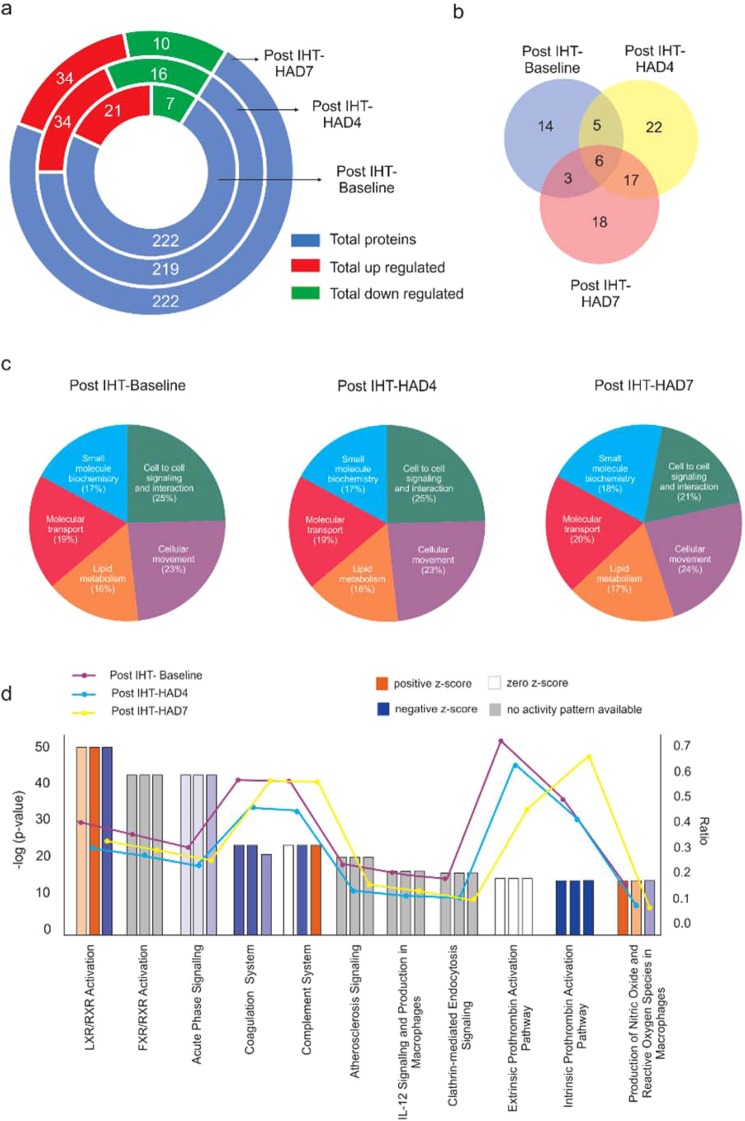
Protein networks in Post-IHT plasma proteome. (**a**) Respective circles of increasing radii depicting total number of identified proteins during LC-MS/MS in Post IHT-Baseline, Post IHT-HAD4 and Post IHT-HAD7 (blue background) and the up-regulated (≥1.5 fold change; red background) and down-regulated (≤0.66 fold change; green background) proteins with respect to Baseline. (**b**) Venn diagram, created using Venny 2.1, showing common proteins between Post IHT-Baseline, Post IHT-HAD4 and Post IHT-HAD7. (**c**) Pie-charts of top 5 GO Molecular and Cellular functions in Post IHT-Baseline, Post IHT-HAD4 and Post IHT-HAD7. (**d**) IPA mined statistically significant Canonical pathways with z-scores and group specific -log (p-values) are shown. Data was analyzed through the use of IPA^35^ (QIAGEN Inc., https://www.qiagenbioinformatics.com/products/ingenuity-pathway-analysis).

Basic understanding of molecular response to intermittent hypoxia (IH) is critical for assessing its effects as a prophylactic intervention regarding high altitude acclimatization. This study was, therefore performed to understand underlying molecular events occurring post intermittent hypoxia training (IHT) at high altitude. A consolidated and global approach was initialized to reveal the origin of molecular changes under intermittent hypoxia. Multiple time points were considered to trace the sequence of molecular events. Global proteomics analysis using iTRAQ based quantitative proteomics showed the presence of 233 different proteins in the plasma of study subjects. However, the mere identity of these proteins in the samples is insignificant hence the comparison of fold change in the expression levels with respect to the sea level (or baseline) samples was performed and a total of 222 proteins (shown in blue) were observed to be differentially expressed in at least one of the experimental group with more proteins up-regulated (shown in red) than down-regulated (shown in green) (Fig. 2a and Supplementary dataset 1). Initial information about the significantly upregulated (>1.5 fold change) and downregulated proteins (<0.66 fold change) showed that Post IHT-Baseline group has 21 upregulated and 7 downregulated proteins, Post IHT-HAD4 group had 34 upregulated and 16 downregulated proteins. Post IHT-HAD7 had 34 upregulated and 10 downregulated proteins (Fig. 2a). There were many proteins common among the experimental groups, which were identified using venny^34^ online tool and it was observed that 6 proteins that were differentially expressed were common among all groups. Also, 14 were unique to post IHT-Baseline, 22 proteins were unique to Post IHT-HAD4 and 18 were unique to Post IHT-HAD7 (Fig. 2b).

We analyzed the protein dataset using Ingenuity Pathway analysis for identifying the networks responding to IHT. The three different groups were comparatively assessed for the top five most dominant molecular and cellular functions. The pie-charts reveal that the major processes are similar across Post-IHT Baseline, Post IHT-HAD4 and Post IHT-HAD7 (Fig. 2c). The molecular and cellular functions that were emphasized and inter-related are Lipid metabolism, Cell to cell signaling and interaction and cellular movement. Remaining two processes, namely Small molecule biochemistry and Molecular transport, derive their relevance from the other functions mentioned above. An initial hypothesis was formed regarding lipid metabolism derived cell signaling events that was associated with redox stress due to hypoxia and possibly modulated the cellular movement observed in inflammatory events.

For the purpose of confirming this hypothesis we performed a comparative analysis of the up- (in orange;) and down- (in blue) regulated pathways across Post IHT-Baseline (first bar from y-axis), Post IHT-HAD4 (second bar from y-axis) and Post IHT-HAD7 (third bar from y-axis). All the network analysis was performed with Baseline expression data as control, and therefore any change thus indicated in the data analysis were indicating a relative alteration with respect to Baseline sample without IHT exposure. Canonical pathway analysis indicated that most significant differentially modulated pathways across groups included LXR/RXR pathway; Acute phase signaling, Coagulation system, Complement system and Production of nitric oxide (NO) and reactive oxygen species (ROS) in macrophages. (Fig. 2d). In case of LXR/RXR pathway, it remains up-regulated in Post IHT-Baseline and Post IHT-HAD4 groups but gets down-regulated in Post IHT-HAD7 group. Coagulation pathway increases its degree of down-regulation with increasing duration at high altitude post IHT. IHT also down-regulates coagulation pathway as observed in Post IHT-Baseline group. Complement pathway is not affected by IHT at sea-level. However, Post IHT-HAD4 group shows down-regulation in this pathway while Post IHT-HAD7 group has up-regulated complement pathway. Another immune system inflammation effector, i.e. Production of NO and ROS in macrophages, is up-regulated by IHT exposure. However, this pathway slowly tends towards down-regulation by 7^th^ day of Post IHT exposure to high-altitude. Overall, when taken together, the trends observed in the last two pathways along with the inhibitory effect on Acute phase signaling response due to IHT indicate that inflammatory signaling is inhibited at high altitude post IHT regimen.

### IHT pre-exposure helps stabilize fatty acid metabolism at high altitude

**Figure 3:**
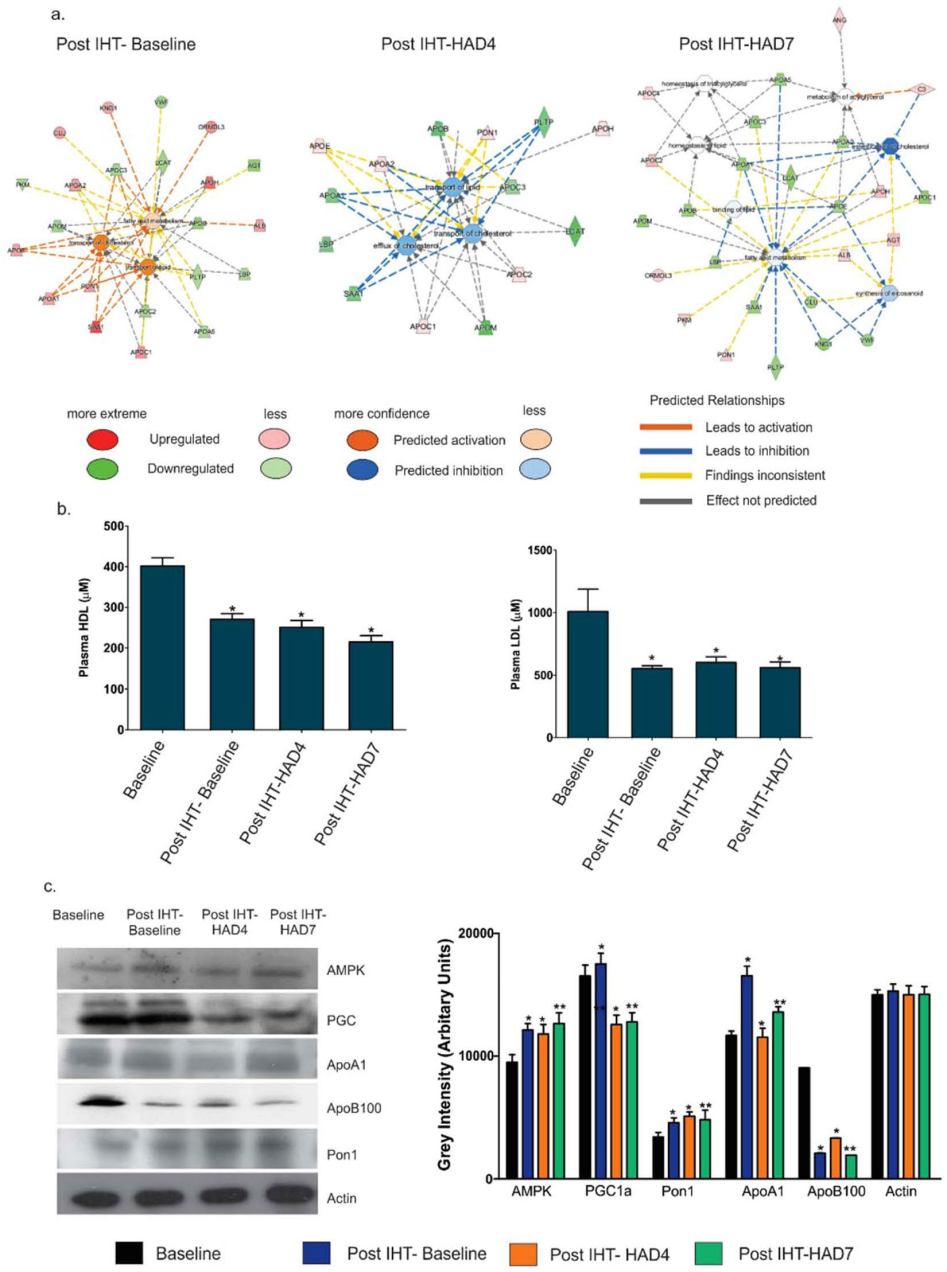
IHT favorably modulates lipid transport and metabolism during subsequent high altitude visit. (**a**) Protein-process networks highlighting lipid metabolism in Post IHT-Baseline, Post IHT-HAD4 and Post IHT-HAD7. The networks were generated through the use of IPA^35^ (QIAGEN Inc., https://www.qiagenbioinformatics.com/products/ingenuity-pathway-analysis). (**b**) Representative bar graph showing HDL-LDL levels (Mean±SEM µM) across Baseline, Post IHT-Baseline, Post IHT-HAD4 and Post IHT-HAD7. (**c**) Immunoblots with densitometry for AMPK, PGC1a, PON1, Apo A1, Apo B100 with actin as loading control. *Represents p < 0.05, ** represents p < 0.01).

Function-protein networks were analyzed with protein expression values overlaid on the relevant proteins in the networks using IPA^35^ (QIAGEN Inc., https://www.qiagenbioinformatics.com/products/ingenuity-pathway-analysis). We focused on processes like fatty acid metabolism, transport of lipid, homeostasis of lipid, synthesis of eicosanoids and efflux of cholesterol that are interlinked to LXR/RXR Activation and energy metabolism with a distant effect on inflammatory signaling (Fig. 3a). We observed that in Post IHT-Baseline group, there is notable up-regulation (indicated by orange) of fatty acid metabolism, transport of lipid and transport of cholesterol. In Post IHT-HAD4, transport of lipid, transport of cholesterol and efflux of cholesterol are down-regulated (indicated by blue). However, even though the duration of hypoxia exposure increased in Post IHT-HAD7, the processes of fatty acid metabolism, homeostasis of lipid and homeostasis of triacylglycerol assume their normal proportions (indicated by no color) while pro-inflammatory processes like synthesis of eicosanoids and esterification of cholesterol are down-regulated (indicated by blue). HDL and LDL levels (Fig. 3b) were also analyzed. HDL levels (Mean±SEM) were 401.63 ± 20.5 µM in Baseline group, 270.79 ± 13.8 µM in Post IHT-Baseline, 250.84 ± 16.9 µM in Post IHT-HAD4 and 215.335200 ± 15.36 µM in Post IHT-HAD7 groups. LDL levels (Mean±SEM) were 1007.31 ± 180.35 µM in Baseline, 553.95 ± 21.68 µM in Post IHT-Baseline, 603.34 ± 42.22 µM in Post IHT-HAD4 and 560.64 ± 45.98 µM in Post IHT-HAD7. It’s evident that although HDL levels remain nearly similar in Post IHT-HAD4 and Post IHT-HAD7, LDL levels spiral downwards in the same two groups. An interesting caveat is that IHT in normoxic conditions tends to increase dyslipidemia while IHT followed by a sufficiently long duration exposure to high altitude tends to help normalize dysregulated lipid metabolism. To further confirm the function-protein networks and investigate the overall direction of energy metabolism, we validated the following proteins: Apo A1, Apo B100, PON1, AMPK and PGC1a (Fig. 3c). AMPK levels were increased Post IHT across all groups (12127.5 AU in Post IHT-Baseline; 11804.3 AU in Post IHT-HAD4 and 12646.3 AU in Post IHT-HAD7) as compared to Baseline (9490.8 AU). This indicates increased fatty acid uptake and metabolism as previously shown in the function-protein networks. PGC1a levels were also increased in Post IHT Baseline group (17505.1 AU vs 16525.8 AU). However, during high altitude exposure as compared to Baseline, the levels of PGC1a decline (12575.2 AU in Post IHT-HAD4 and 12773.6 AU in Post IHT-HAD7). Since PGC1a is considered the master regulator of mitochondrial biogenesis^38,39^, IHT exposure in normoxic conditions help augment mitochondrial density. Even though this process is halted during high altitude exposure, possibly due to limited aerobic respiration, the advantages of increased mitochondrial density gained prior to hypoxic exposure is worthwhile even beyond the context of a high-altitude visit. Apo A1, a component of HDL and helpful in efflux of fats from cells for their transport, was also validated. Its levels are increased in Post IHT-Baseline group (16548.86 AU vs 11674.5 AU in Baseline). In Post IHT-HAD4, Apo A1 levels decline (11523.490 AU) before increasing again in Post IHT-HAD7 (13582.81 AU). Apo A1 is very important since it’s considered a predictor of cardiovascular disease. Apo B100, a constituent of LDL/VLDL and an indicator of their levels, was also immunoblotted. Apo B100 levels were found to be decreased in Post IHT-Baseline (2103.13 AU vs 9037.93 AU in Baseline) before recovering in Post IHT-HAD4 (3329.74 AU) and finally decreasing again in Post IHT-HAD7 (1928.54 AU). Apo A1 and Apo B100 levels corroborated the trends of HDL and LDL graphs. PON1, also a major anti-atherosclerotic protein in HDL, keeps increasing Post IHT exposure in all groups (4576.380 AU in Post IHT-Baseline; 5110.14 AU in Post IHT-HAD4 and 4831.70 AU in Post IHT-HAD7) vs Baseline (3422.68 AU). Since dysregulation of lipid metabolism is surely halted due to IHT regimen prior to high altitude stay, we focused on the inflammatory processes that are linked to functions like lipid homeostasis, esterification of cholesterol and synthesis of eicosanoids.

### IHT exposure prior to high-altitude stay activates anti-inflammatory processes via priming inflammatory proteins

**Figure 4:**
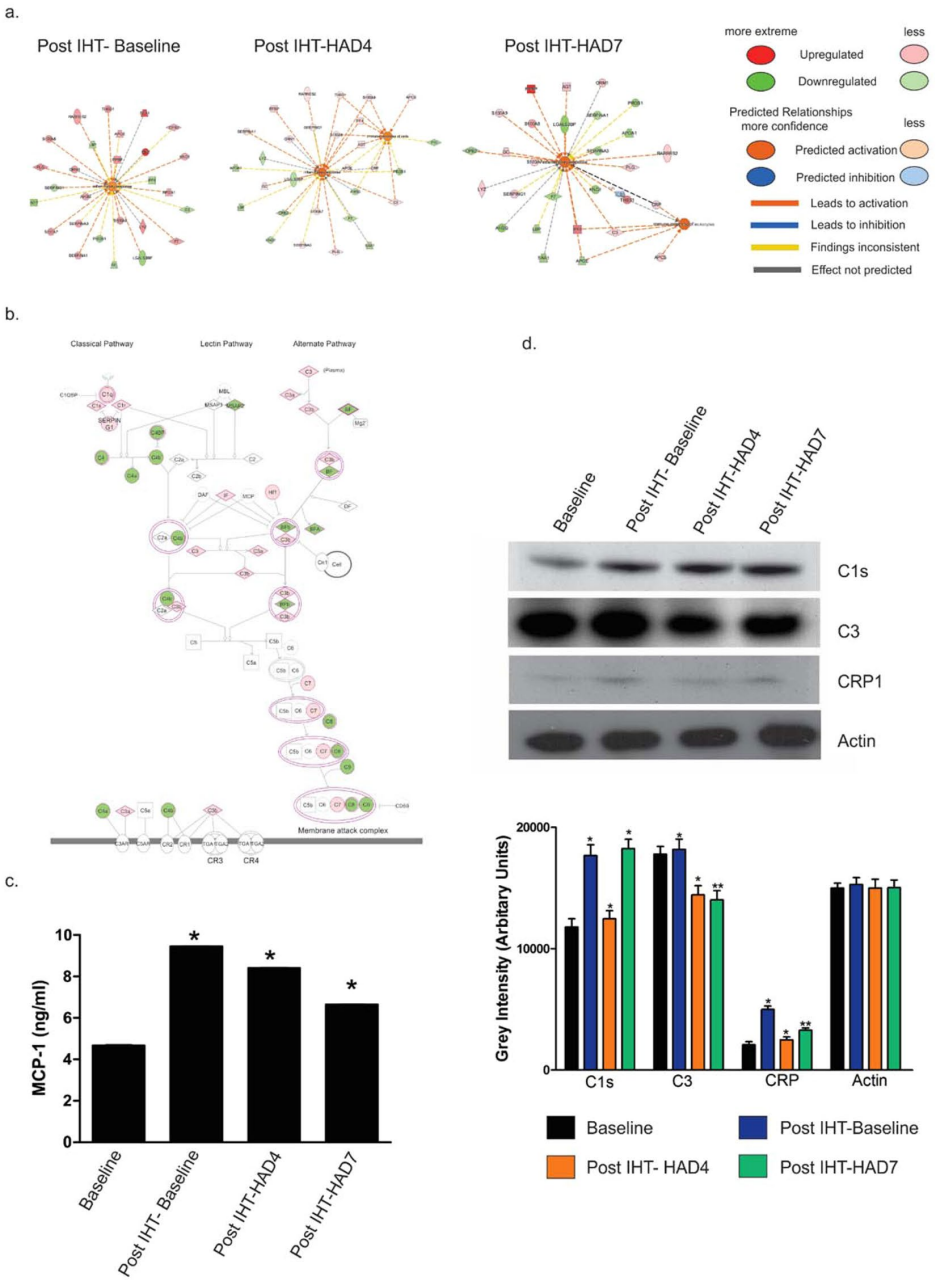
IHT causes subdued inflammatory processes at high altitude. (**a**) Protein-process networks highlighting inflammatory processes in Post IHT-Baseline, Post IHT-HAD4 and Post IHT-HAD7. (**b**) IPA Canonical pathway for Complement system with overlaid protein expression data. Protein up-regulation is shown as orange while down-regulation is shown as green. The networks (in a and b) were generated through the use of IPA^35^ (QIAGEN Inc., https://www.qiagenbioinformatics.com/products/ingenuity-pathway-analysis). (**c**) Monocyte chemotactic protein 1 (MCP-1) concentrations (mean ± SEM ng/ml) in plasma of Baseline, Post IHT-Baseline, Post IHT-HAD4 and Post IHT-HAD7 (* represents p-value < 0.05). (**d**) Mean pixel intensities of immunoblots of C1s, C3 and CRP with Actin as loading control (* represents p < 0.05, ** represents p < 0.01).

It is widely stated that high altitude exposure causes redox stress and inflammation^40–42^. Thus, IHT, as a prophylactic intervention against hypobaric hypoxia or high altitude, needs to be assessed for its ability to modulate inflammation. Function-protein networks were again analyzed using IPA (QIAGEN Inc., https://www.qiagenbioinformatics.com/products/ingenuity-pathway-analysis). However, the focus was shifted to inflammatory response and immune response networks (Fig. 4a). It was observed that across all groups, post IHT, inflammatory response network was up-regulated. The inflammatory response consisted of both anti-inflammatory and pro-inflammatory molecules. We trawled through the relevant proteins in our dataset involved in classical and alternate complement pathways overlaying expression data onto the pathways (Fig. 4b). Lectin pathway was sidelined as our experiment did not involve any bacterial infection. As a general indicator of inflammatory processes was required, we analyzed Monocyte Chemotactic Protein-1 (MCP-1) levels using ELISA (Fig. 4c). MCP-1 was activated by IHT, increasing initially but declining in high altitude groups (9.45 ng/ml in Post IHT-Baseline; 8.41 ng/ml in Post IHT-HAD4; 6.65 ng/ml in Post IHT-HAD7 vs 4.67 ng/ml in Baseline). C1s, CRP and C3 immunoblots were also analyzed (Fig. 4d). C1s, a component of C1 complex, increases in Post IHT-Baseline group (17685.67 AU vs 11791.97 AU in Baseline) before decreasing in Post IHT-HAD4 (12469.6 AU). Thereafter, C1s levels are heightened in Post IHT-HAD7 group (18255.02 AU). In line with C1s, C3 levels also increase in Post IHT-Baseline group (18185.4 AU vs 17792.8 AU in Baseline) before decreasing nearly equally in both Post IHT-HAD4 (14433.7 AU) and Post IHT-HAD7 groups (14020.8 AU). CRP levels were observed to increase in Post IHT-Baseline (5004.06 AU vs 2087.95 AU Baseline) before decreasing in Post IHT-HAD4 (2491.52 AU) and slightly incrementing in Post IHT-HAD7 (3280.97 AU). Thus, IHT is causing inflammation which is subdued by anti-inflammatory processes. This is in line with the canonical pathway data showing de-activation of oxidative burst in macrophages in Post IHT-HAD7. The activated anti-inflammatory processes become a boon at high altitude where pro-inflammation networks are subdued. The overall conclusion that can be drawn from these and the results shown in previous section is that IHT exposure prior to high-altitude exposure helped augment lipid homeostasis and finally subdued pro-inflammatory signaling during high altitude stay.

**Figure 5:**
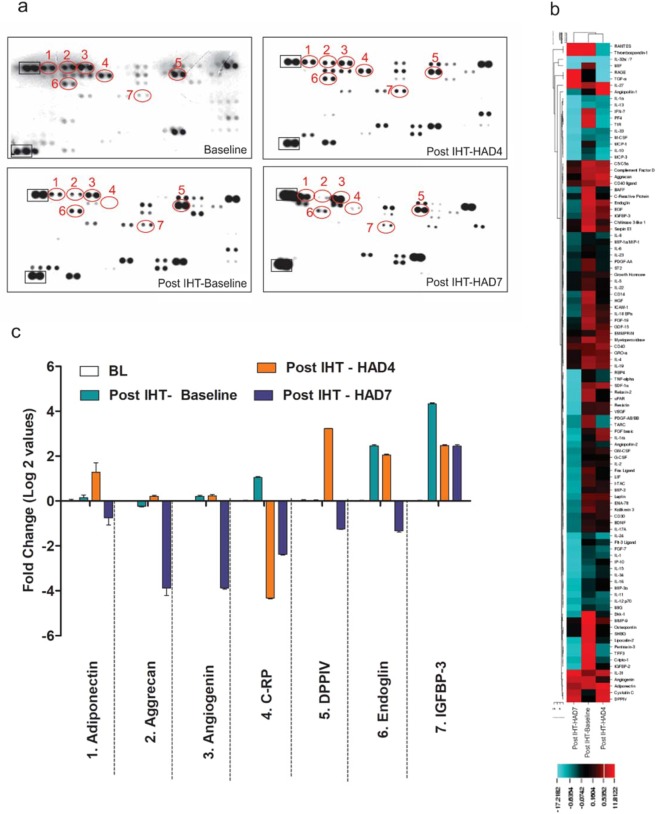
Cytokine array for assessment of inflammatory signaling. (**a**) Representative image of pre-coated cytokine array with seven most perturbed cytokines encircled in red. (**b**) Heat-map with hierarchical clustering of experimental groups based on normalized fold change values based on cytokine array. (**c**) Bar graphs representing top 7 most perturbed cytokines as log 2 (fold change).

To confirm subdued inflammatory signaling using a second line of experiments, we further analyzed a cytokine array (Fig. 5; supplementary dataset 2) for the stated experimental groups. Most of the cytokines were observed to be down-regulated in Post IHT-HAD7 (Fig. 5b,c). A clear trend is observed wherein cytokines in Post IHT-Baseline start showing higher levels and more cytokines with higher levels are observed in Post IHT-HAD4 before a reversal in Post IHT-HAD7. The results of the cytokine array again proved that pro-inflammatory events were primed by IHT regimen. However, this priming caused the organism to subdue the inflammatory events during high-altitude stay. Since the cause of dysregulated lipid homeostasis and inflammatory processes is redox stress at high altitude, we hypothesized that mild redox stress provided by IHT regimen caused the individuals’ redox homeostasis machinery to already be primed ahead of high-altitude exposure. To assess the veracity of this hypothesis, we analyzed the redox parameters like MDA and levels of antioxidant enzymes like glutathione.

### IHT causes redox stress which subsequently enables better management of redox stress during high altitude stay at molecular level

**Figure 6:**
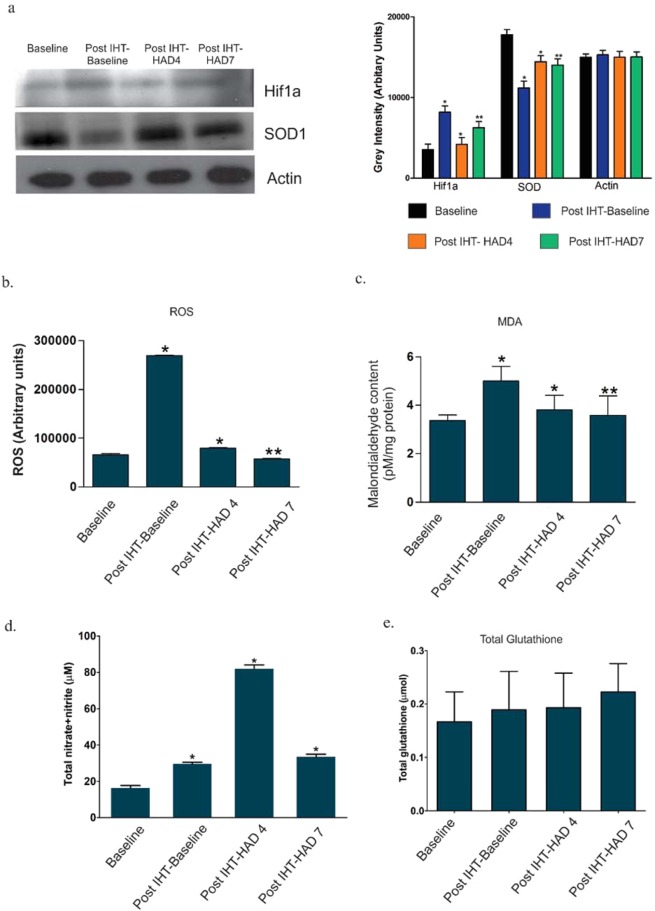
Modulation of redox processes by IHT and subsequent effects at high altitude. (**a**) Mean pixel intensity of immunoblots of HIF1a and SOD with Actin as loading control (*represents p < 0.05, ** represents p < 0.01). (**b**) ROS levels (AU), (**c**) MDA/TBARS levels (pM/mg protein), (**d**) Total NOx levels (µM/mg protein) and (**e**). Total glutathione levels (µmol) in Baseline, Post IHT-Baseline, Post IHT-HAD4 and Post IHT-HAD7 (* represents p < 0.05, ** represents p < 0.01).

Redox stress is considered the underlying unifying molecular event that when not managed by the individuals’ redox homeostatic mechanisms causes high altitude associated illnesses like AMS and HAPE^40,43^. Since our findings show prior IHT in good light for favorably modulating energy metabolism and controlling inflammation during high altitude stay, we analyzed if IHT was associated with favorably modulating the redox machinery of the individual during high altitude stay. HIF1a, the principal component of the cellular machinery to respond to hypoxia induced redox stress was analyzed using immunoblots (Fig. 6a). Its levels were observed to be increased at Post IHT- Baseline (8184.037 AU vs 3555.89 AU in Baseline) before declining in Post IHT-HAD4 (4214.44 AU) and recovering notably in Post IHT-HAD7 group (6257.38 AU). Superoxide dismutase, a central antioxidant protein, was also immunoblotted (Fig. 6a). Although its levels decrease in Post IHT- Baseline group (11185.4 AU vs 17792.8 AU in Baseline), Post IHT-HAD4 (14433.7 AU) and Post IHT-HAD7 (14020.8 AU) groups show augmentation. We performed ROS, MDA (malondialdehyde), GSH (glutathione) and nitric oxide (NOx) estimation (Fig. 6b–e) in the plasma samples. ROS levels increased due to IHT in Post IHT-Baseline (269075 AU vs 65805 AU in Baseline) but were lower in Post IHT-HAD4 (79110 AU) and Post IHT-HAD7 (57465 AU) groups. MDA levels also showed a similar trend. Post IHT-Baseline levels (Mean±SEM = 5 ± 0.6 pM/mg protein) increased significantly as compared to Baseline levels (3.37 ± 0.23 pM/mg protein). However, MDA levels were near Baseline in Post IHT-HAD4 (3.80 ± 0.6 pM/mg protein) and Post IHT-HAD7 (3.59 ± 0.8 pM/mg protein) groups. NOx levels also increased in Post IHT-Baseline (Mean±SEM = 63.83 ± 0.98 µM/mg protein vs 18.95 ± 1.38 µM/mg protein) before decreasing in Post IHT-HAD4 (33.65 ± 2.13 µM/mg protein) and Post IHT-HAD7 (23.19 ± 1.43 µM/mg protein) groups. GSH levels on the other hand, showed a continuous but meagre rise across all groups Post IHT (Mean±SEM: 0.189 ± 0.072 µmol in Post IHT-Baseline; 0.193 ± 0.065 µmol in Post IHT-HAD4 and 0.222 ± 0.053 µmol in Post IHT-HAD7) with respect to Baseline (0.167 ± 0.056 µmol) group. Thus, mild IHT regimens similar to the one described in this manuscript can be termed a beneficial prophylactic for high-altitude stays extending till seven days. This is based on the fact that by inducing mild redox stress, dysregulated lipid metabolism and inflammatory signaling, IHT primes individuals’ redox and energy homeostasis mechanisms favorably for high altitude stays.

### Protein nitrosylation and Protein carbonylation are affected by both IHT and high-altitude hypoxia

**Figure 7:**
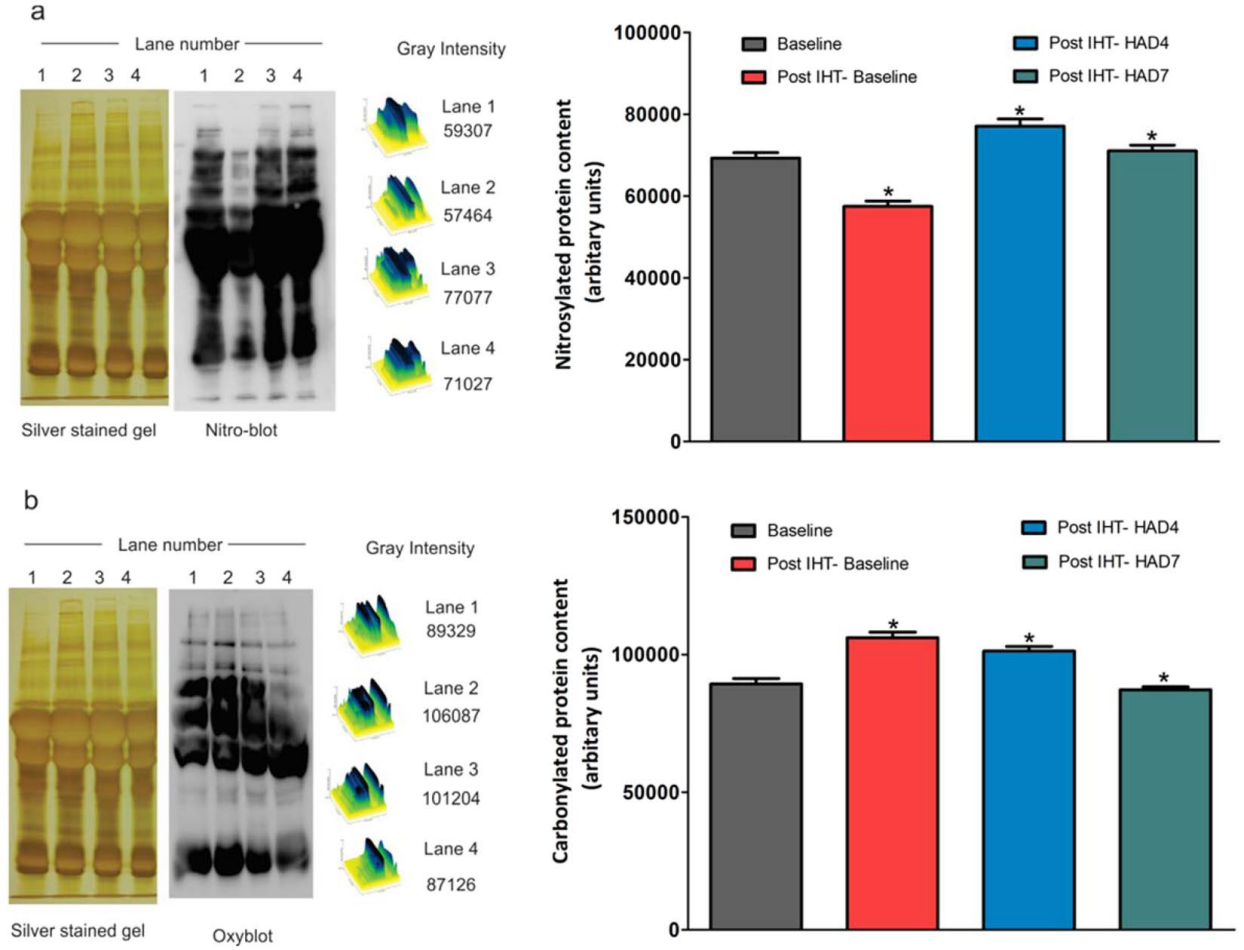
Effect of IHT on protein S-Nitrosylation and protein carbonylation. (**a**) Representative nitro-blot of Baseline (BL), Post IHT-BL, Post IHT-HAD4 (high altitude day 4) and Post IHT-HAD7(high altitude day 7). Silver stained gel represents loading control. (**b**) Representative oxy-blot showing levels of protein carbonylation in Baseline (BL), Post IHT-BL, Post IHT-HAD4 (high altitude day 4) and Post IHT-HAD7 (high altitude day 7). Silver stained gel represents loading control.

Protein nitrosylation and carbonylation were observed to be important post-translational modifications in context of high altitude hypoxia induced redox stress^44,45^. Nitrosylation and carbonylation are involved in negative cross-talk as one inhibits the other^30^. A general trend of protein nitrosylation (Fig. 7a) shows nitrosylated proteins decreasing in Post IHT-BL. However, subsequent high altitude days (Post IHT-HAD4 and Post IHT-HAD7) show increased levels of nitrosylation. We also analyzed carbonylation levels to observe the effects of IHT (Fig. 7b). Oxy-blot shows an increase in Post IHT-BL protein carbonyl content. However, at subsequent high altitude exposure days, i.e. Post IHT-HAD4 and Post IHT-HAD7, there are sequential decreases in carbonyl content. This is in contrast to nitrosylation trends and further confirms our previous *in vitro* observations regarding negative cross-talk between nitrosylation and carbonylation.

Prophylaxis with IHT consisting of repeated exposures to 12–14% FIO_2_ proved beneficial for high-altitude stays as long as seven days. This was attributable to IHT induced priming of redox and energy homeostasis mechanisms as well as anti-inflammatory processes subduing the adverse molecular effects of high altitude hypoxia. Protein nitrosylation and carbonylation trends are in line with the previous findings and are additional evidence of the benefits of IHT based on the benefits of increased NO products at high altitude^44,46,47^.

## Discussion

The pros and cons of intermittent hypoxia administration have been a subject of active investigation in the recent past, primarily due to its clinical advantage. Our study is in line with the understanding of underlying cellular events of intermittent hypoxia induced changes in the human proteome and their implications during high altitude exposure. The existing experimental regimen was chosen based on the previous studies which evaluated human performance at FIO_2_ values close to 12-13%^48–51^. In this study, experimental data provided highly relevant network insights, in particular about the redox events, lipid metabolism and inflammatory axis modulations.

The intermittent hypoxia in our study was for a period of short duration (of simulated hypoxia) followed by a prolonged assessment at actual high-altitude condition. This study was therefore, different from several previous studies which were limited to either separate long-term or short-term hypoxia regimen. Dumitrovici *et al*. state that “Controversial effects of intermittent hypobaric hypoxia (IHH) such as cardiac damage or cardiac protection are still mysterious. It is unclear if short-term and long-term IHH challenges exert different changes of the pro-oxidant/antioxidant balance in the heart and throughout the body”^52^ indicating short term and long term effects of IH need to be explored in greater detail. Basset *et al*. found that the levels of hematocrit, hemoglobin, platelet number\erythropoietin concentration and red blood cell count was increased upon short-term normobaric hypoxia exposure in highly trained athletes but without any changes in physical performance^53^. A long-term hypoxia study on soldiers by Prommer *et al*. suggests that higher changes in arterial oxygen mainly resulted from augmented hemoglobin mass and at altitude also from increased arterial oxygen-saturation, however, the acclimatization to long-term intermittent hypoxia substantially increases SaO_2_, but has no beneficial effects on physical performance either at altitude or at sea level^54^. Even in neonatal development, benefits of IHT have been showcased by Zhang and colleagues. Their study showed improved performance of mice in water maze and 8-arm radial maze tasks upon neonatal IHT exposure^55^. It is now increasingly recognized that physiological outcomes of IH widely varies depending on pattern, intensity and duration of application^10^. Our study is, in fact a combination of such situations where we have combined mild IHT with acute high-altitude exposure providing a network wherein redox events are associated with changes in lipid metabolism and inflammatory signaling.

About 233 proteins were identified, however many proteins were not detected in other groups, perhaps due to threshold of detection and therefore limited the comparison of those proteins among experimental groups. We, therefore relied mainly on those proteins which were common among all groups (~222 proteins). A comprehensive list of MS data was sequentially processed and bioinformatics tools such as IPA (QIAGEN Inc., https://www.qiagenbioinformatics.com/products/ingenuity-pathway-analysis) were used to unravel the underlying information in mass spectrometry data.

**Figure 8:**
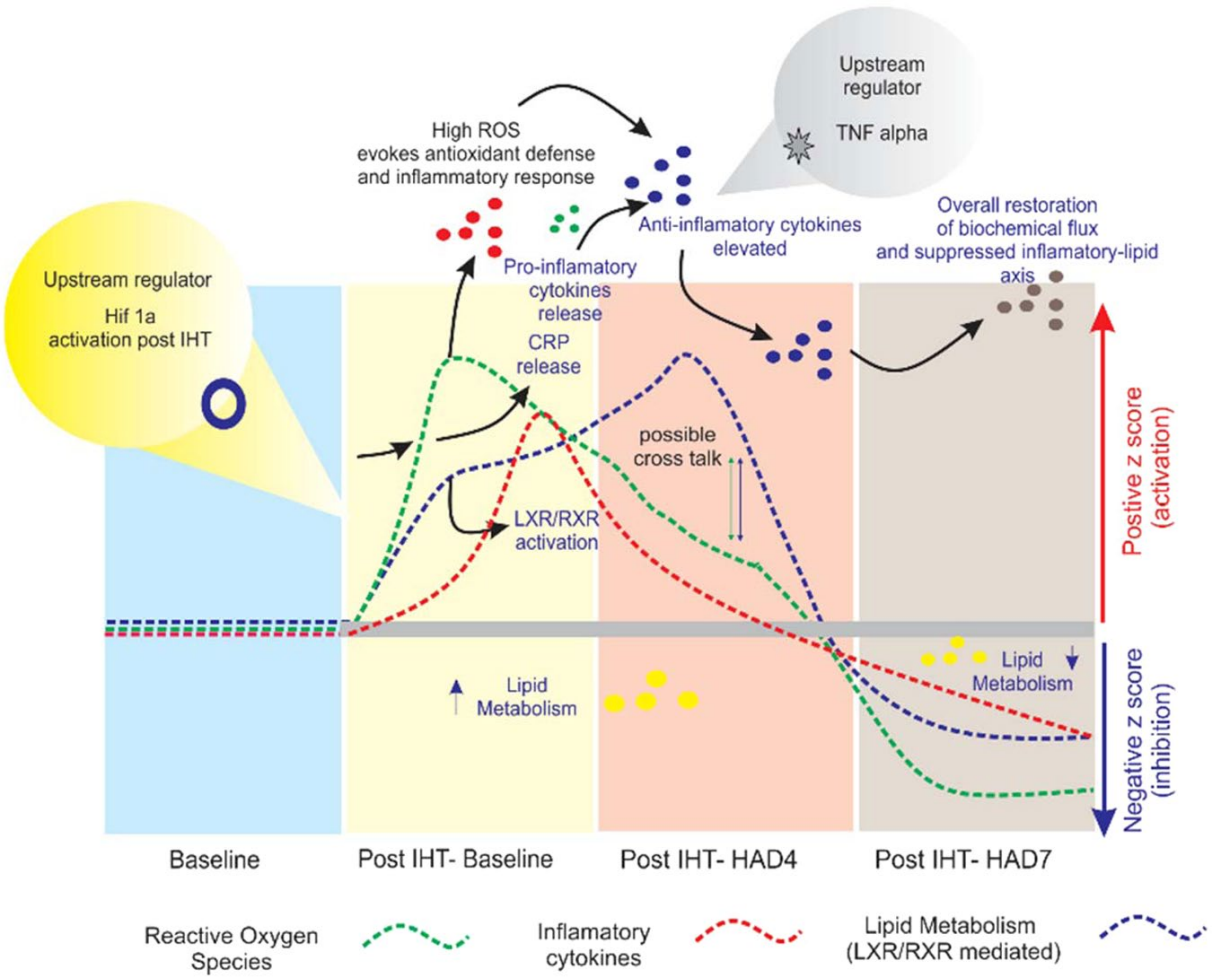
IHT modulates the trinity of lipid metabolism, redox homeostasis and inflammation resulting in beneficial effect during high altitude exposure. IHT at sea-level incites reactive oxygen species (ROS) generation, dysregulation of LXR/RXR mediated lipid metabolism and inflammatory cytokines via eicosanoids and inositol phospholipids (inflammatory by-products of lipid metabolism). Increased HIF1a expression and activity occurs due to IHT. These processes trigger their management systems, e.g. redox stress triggers redox homeostasis proteins like SOD and GSH. This leads to improved anti-oxidant and anti-inflammatory responses during post IHT high-altitude exposure. At high altitude day 4 (Post IHT-HAD4), ROS and inflammatory cytokines decrease. Next, we observed a cross-talk based lipid metabolism modulation so that its activity is closer to Baseline at high altitude day 7 (Post IHT-HAD7). Hence, redox stress, lipid metabolism and subsequently inflammatory processes are normalized to Baseline levels.

Preliminary canonical pathway analysis indicated disruption of lipid metabolism and inflammatory processes being activated in Post IHT-Baseline group with respect to baseline (Fig. 2d). LXR/RXR pathway was prominent and activated very strongly in Post IHT-HAD4 while inhibited in Post IHT-HAD7 group. The primary role of LXR/RXR pathway is in lipid metabolism based energy homeostasis. The liver X receptor (LXR) is a member of the nuclear receptor family of transcription factors and is closely related to nuclear receptors such as the PPARs, FXR and RXR. Liver X receptors (LXRs) are known to be important regulators of cholesterol, fatty acid, and glucose homeostasis^56–59^. Recently, Hong *et al*. have explored the opportunities for drug discovery based on LXRs^60^, however its role in hypoxic acclimatization is still unclear. Alteration in this major pathway appears to influence lipid metabolism. Another important phenomenon that links with RXR down regulation in Post-IHT HAD7 group is the de-activation of acute phase response (Fig. 2d). Interestingly, a correlation between down-regulation of LXR/RXR and negative acute phase response pathways in colon adenocarcinoma has been revealed by proteomics and bioinformatics analysis^61^. Additionally, LXR-mediated activation of macrophage via stearoyl-CoA desaturase was also studied^62^. These recent discoveries suggest a close interplay of inflammatory axis and lipid metabolism. The present study adds to this growing tree of evidence. Coagulation system was inhibited by IHT and it remained down-regulated during high altitude stay. This is a boon for those at risk of thrombosis, a major health risk at altitude^63,64^. Complement system was down-regulated in Post IHT-HAD4 group before being up-regulated in Post IHT-HAD7 (Fig. 8). Activation of complement system at high altitude is termed a normal occurrence. However, its delayed onset after seven days of high-altitude stay, in addition to the fact that anti-inflammatory cytokines were also activated, is a beneficial aspect of IHT (Fig. 8). When coupled with the down-regulation of other immune processes like Production of NO and ROS by macrophages in Post IHT-HAD7 group, it shows mild IHT to be an effective modulator of both energy metabolism and immune/inflammatory processes at high altitude. When we compare it to previous similar studies by Padhy *et al*.^32^, the 4^th^ day of exposure shows definitive differences in inflammation and redox stress at similar altitude. NOx levels are much higher at the 4^th^ day of altitude exposure in Post-IHT groups as compared to non-IHT groups. Even carbonylation trends suggest lower carbonyl content in plasma proteome of Post IHT-HAD4 as compared to non IHT HAD4 from the previous study.

Metabolic consequences of intermittent hypoxia have been understood to a greater depth in sleep apnea, where studies suggest that IH is independently associated with metabolic dysfunction, including dyslipidemia and insulin resistance. IH is speculated to affect glucose metabolism by inducing sympathetic activation, increasing systemic inflammation, increasing counter-regulatory hormones and fatty acids, and causing direct pancreatic beta-cell injury^65^. In another study by O’Donnell *et al*., it was found that an exposure to intermittent hypoxia produced whole-body insulin resistance in lean healthy mice and reduced glucose utilization in oxidative muscle fibers, but did not cause a change in hepatic glucose output. Furthermore, the increase in insulin resistance was not affected by blockade of the autonomic nervous system. Therefore, it is noteworthy to comment that intermittent hypoxia can cause acute insulin resistance in otherwise lean healthy animals^66^. However, these reports employ or study severe IH of a chronic and intense nature. Previous reports have made amply clear that such severe IH only brings unpleasant after-effects like oxidative stress induced systemic inflammation underlying a number of detrimental cardiovascular, respiratory, metabolic, and cognitive outcomes^1,2,67–70^. Other studies have pinpointed the role of IH pattern, intensity and duration of application to change the spectrum of its effects to beneficial^10,71–73^.

We also observed metabolic perturbations pertaining to lipid metabolism (Fig. 3a). Lipid metabolism and transport were activated immediately in Post IHT-Baseline but they were inhibited strongly at Post IHT-HAD4 group before normalizing at Post IHT-HAD7 (Fig. 3). Some recent studies have also shown similar findings, a study by Drager *et al*. on the metabolic consequences of intermittent hypoxia has revealed dyslipidemia after intermittent hypoxia^22,74^. Although, as a limitation, the clear underlying mechanism of this change was not deciphered in this study, it has been widely reported that high altitude stays usually cause anorexia, hypophagia and nausea at least in the initial period of stay^75,76^. Also, some authors have pointed out the selective fat loss during high altitude ascents in mountaineers with duration of exposure exceeding seven days^77,78^. Future studies may elucidate this aspect completely. However, the recovery of Baseline levels of lipid metabolism (Fig. 8) by seventh day of exposure showed that prophylaxis with mild IHT led to a favorable outcome at high altitude. HDL and LDL fractions were also plotted (Fig. 3b). Although no significant increase was measured in HDL levels at altitude, the LDL levels remained low at high altitude days (Post IHT-HAD4 and Post IHT-HAD7) with the lowest levels of LDL reported at seventh day of altitude exposure. Related apolipoproteins like Apo A1 and Apo B100 were also immunoblotted (Fig. 3c). Apo A1, a component of HDL particles, after decreasing in Post IHT-HAD4 group increased in Post IHT-HAD7 group. PON1, another anti-oxidant protein component of HDL, kept increasing after Post IHT exposure across all groups. This corresponds with increased anti-inflammatory effect of HDL. Apo B100, a component of LDL/VLDL fractions, decreased in Post IHT-Baseline but increased in Post IHT-HAD4. This increase was however arrested at Post IHT-HAD7. When taken together, this indicates that due to IHT LDL/VLDL levels were decreased while anti-inflammatory capacity of HDL increased at high altitude (Fig. 8). At the upstream level the role of key metabolic switches such as AMPK, PGC1a and PON1were also studied. AMPK, which activates metabolic processes resulting in uptake of fatty acids, was up-regulated due to IHT in all Post IHT groups with respect to baseline. AMPK is also reported as a master switch of metabolic regulation and reactive oxygen species metabolism which is known from several studies to perturb the proteome profile during hypoxic exposures^79–81^. PGC1a, the master regulator of mitochondrial biogenesis and essential for energy homeostasis, was also up-regulated due to IHT. Its levels declined upon high altitude stay possibly due to lack of aerobic respiration. However, increased mitochondrial biogenesis even after the process is down-regulated will provide beneficial after effects as seen in lipid homeostasis and controlled inflammation. PON1, known to be anti-atherosclerotic protein in HDL and involved in innate immune response to gram negative bacteria^82,83^, was also found to increase in all Post IHT groups (Fig. 3c). Taken together these findings indicate an overall systemic recalibration towards normalized lipid and energy metabolism at Post IHT-HAD7 (Fig. 8). Function-protein network analysis showed that some of the proteins were common between lipid metabolism dysregulation and inflammatory response like kininogen 1 (KNG1), serum amyloid A1 (SAA1), Apo A1, Apo E and C3 (Figs. 3a & 4a). Thus, our next focal investigation centered on assessing whether normalization of inflammatory processes also occurred at high altitude due to IHT.

Firstly, we assessed the overall progress of the complement pathways by validation of MCP-1, C1s, C3 and CRP (Fig. 4c,d). The overall direction of the inflammatory response was assessed to be towards a subdued inflammatory signaling response with major hints of anti-inflammatory processes, particularly those that are indirect (e.g. low LDL levels in Post IHT-HAD7) in nature. In order to delve deeper into inflammatory axis and its relation with intermittent hypoxia, cytokine array was performed (Fig. 5). The natural conclusion that can be drawn from cytokine array results is the up-regulation of most of the cytokines at Post IHT-Baseline and gradual dampening culminating in mostly down-regulated cytokines through Post IHT-HAD4 at Post IHT-HAD7 groups. This supports the notion that somehow inciting inflammatory processes at Baseline using IHT was helping systemic anti-inflammatory signaling, particularly in context of cytokine cascades, during subsequent exposure to high altitude (Fig. 8). However, the exact signaling cascades responsible for this effect remain elusive and can be the subject matter of future studies.

From this study, the downstream events occurring Post IHT in HAD4 and HAD7 were clear, but the upstream regulator still remains unclear. In our attempt to understand this, we estimated the master regulator of hypoxic response, HIF1a^84^. Since all hypoxic responses can be traced to creation of oxygen and nitrogen radicals in the mitochondria^85^, IH, i.e. low oxygen percentages, was also causing lipid metabolism dysregulation and inflammatory signaling based on its ability to trigger redox stress. Thus, elevated reactive oxygen species levels and nitrogen species was tested directly using biochemical assay and observed to be consistent with the predictions made by network analysis of global proteomic data. Central anti-oxidant proteins like SOD and GSH were also observed to be up-regulated. Redox post-translational modifications, particularly nitrosylation and carbonylation, were also assessed. Their trends were in accordance with previous findings^30^. NOx estimation (Fig. 4.6d) showed greater total NOx in Post IHT-BL, Post IHT-HAD4 and Post IHT-HAD7 when compared to Baseline. Thus, we measured total nitrosylation in plasma proteome (Fig. 7a) and observed that it increased in Post IHT-HAD4 and Post IHT-HAD7. Total carbonyl content was also estimated using oxy-blots (Fig. 7b). This is the first instance of anti-trends and possible cross-talk being demonstrated between nitrosylation and carbonylation *in vivo* as an after-effect of IHT prophylaxis. Kumar and Prabhakar have enumerated chronic intermittent hypoxia induced phosphorylation using gel-based immunostaining methods^86^. Hence, this study provides a fresh insight into IH associated post-translational modifications.

Previously reported high altitude exposure after-effects like dysregulated energy metabolism^87^, redox stress^88^ and inflammation^89,90^ were prevented due to mild IHT. This shows that, prophylaxis with IHT (FIO_2_ ranging between 12-14%) benefits subsequent high-altitude exposure even in case of rapid ascent. Another central regulator of inflammatory axis that was predicted in proteomics data was TNF alpha^91^ (Supplementary figure S1; Supplementary dataset 2), which was also validated during cytokine array and found to be upregulated during IHT induced cytokine surge. However, this mild IH challenge caused a priming of energy homeostasis molecules like AMPK, activation of systemic anti-inflammatory signaling (demonstrated by falling levels of C3, CRP and MCP-1 in Post IHT groups) and augmentation of antioxidant molecules like SOD and GSH. This ensured that previously reported molecular perturbations during high altitude exposure like dysregulated energy metabolism^87^, redox stress^88^ and inflammation^89,90^ were subdued. Thus, prophylactic interventions using mild IHT regimens can be termed beneficial to those who in the near future undertake a sojourn to high altitude areas.

## Conclusion

We observed IHT to cause a spike in redox stress (via increased ROS), increased inflammatory cytokine activation and production and abnormal LXR/RXR activation mediated lipid metabolism near sea-level. This caused HIF1-alpha elicited transcriptional modulation. The increased ROS levels, pro-inflammatory cytokines and particularly CRP led to activation of systemic antioxidant proteins and TNF-alpha led modulation of an anti-inflammatory cytokine response. Thus, we observed that at high altitude exposure day 4 (Post IHT-HAD4) the levels of ROS and inflammatory cytokines decrease. As per the findings of a previous study^32^, the anti-inflammatory and anti-oxidant responses were observed at high altitude exposure day 7. Somehow, IHT prompts the dysregulated lipid metabolism to remain closer to Baseline levels at Post IHT-HAD7 while ROS and cytokine levels continue to decline (Fig. 8). Although there is clear suppression of the inflammatory lipid axis in Post IHT-HAD7 group, the exact mechanistic details need to be investigated.

This study provides new facets to the molecular effects of intermittent hypoxia and poses some new questions about lipid metabolism dysregulation, redox stress and inflammation. Although there is indication of cross talk and common proteins between the trinity of redox homeostasis, lipid metabolism and inflammation during IH and high-altitude exposure, the exact mediators within this trinity are left for future studies to explore beyond high altitude (and hypoxia) further into the surge of metabolic syndrome and its co-morbidities like obesity and diabetes.

### Study limitations

The present study has a few limitations. The most important limitation is the small sample size (n = 40). The second caveat is all volunteers were young, healthy males following nearly identical daily schedules. Thus, factors like gender, lifestyle choices and aging are not accounted for. Finally, we did not comparatively assess all three altitude zones (high, very high and extreme altitude zones). Individuals at 3500 m were assessed which falls in the high altitude category. Thus, whether IHT will be beneficial at even higher altitudes is still an open question. Another limitation is the unavailability of IHT logistics to the masses due to cost and technical expertise. This must be resolved by providing cost-effective IHT equipment for mass use.
